# Lonidamine and domperidone inhibit expansion of transformed cell areas by modulating motility of surrounding nontransformed cells

**DOI:** 10.1016/j.jbc.2022.102635

**Published:** 2022-10-21

**Authors:** Megumi Aoyama, Kosuke Ishikawa, Shuntaro Nemoto, Hiroyuki Hirano, Nobumoto Watanabe, Hiroyuki Osada, Shinya Watanabe, Kentaro Semba

**Affiliations:** 1 Department of Life Science and Medical Bioscience, School of Advanced Science and Engineering, Waseda University, Tokyo, Japan; 2 Japan Biological Informatics Consortium (JBiC), Koto-ku, Tokyo, Japan; 3 RIKEN Center for Sustainable Resource Science, Wako, Saitama, Japan; 4 Department of Pharmaceutical Sciences, University of Shizuoka, Suruga-ku, Shizuoka, Japan; 5 Translational Research Center, Fukushima Medical University, Fukushima, Japan

**Keywords:** proliferation, anticancer drugs, epithelial cells, oncogene, drug screening, cell motility, lonidamine, domperidone, CA, constitutive active, CIL, contact inhibition of locomotion, DMSO, dimethyl sulfoxide, DN, dominant-negative, DOX, doxycycline, DPD, domperidone, HRP, horseradish peroxidase, LND, lonidamine, MPC, mitochondrial pyruvate carrier, NMuMG, normal murine mammary gland

## Abstract

Cancer cells intrinsically proliferate in an autonomous manner; however, the expansion of cancer cell areas in a tissue is known to be regulated by surrounding nontransformed cells. Whether these nontransformed cells can be targeted to control the spread of cancer cells is not understood. In this study, we established a system to evaluate the cancer-inhibitory activity of surrounding nontransformed cells and screened chemical compounds that could induce this activity. Our findings revealed that lonidamine (LND) and domperidone (DPD) inhibited expansion of oncogenic foci of *KRASG12D*-expressing transformed cells, whereas they did not inhibit the proliferation of monocultured *KRASG12D*-expressing cells. Live imaging revealed that LND and DPD suppressed the movement of nontransformed cells away from the attaching cancer cells. Moreover, we determined that LND and DPD promoted stress fiber formation, and the dominant-negative mutant of a small GTPase RhoA relieved the suppression of focus expansion, suggesting that RhoA-mediated stress fiber formation is involved in the inhibition of the movement of nontransformed cells and focus expansion. In conclusion, we suggest that elucidation of the mechanism of action of LND and DPD may lead to the development of a new type of drug that could induce the anticancer activity of surrounding nontransformed cells.

Proper regulation of cell proliferation, growth, and cell death is essential for maintaining the normal function and morphology of organs in the body. Accumulating genetic and epigenetic aberrations involved in this regulation result in the development and spread of tumors. Clones with acquired mutations in cancer driver genes expand in phenotypically noncancerous tissues without forming an apparent tumor. This phenomenon, called “clonal expansion,” has been observed in various tissues and organs, including the skin and the esophagus (1). The significant overlaps of genetic mutations in noncancerous and corresponding cancerous tissues support the possibility that the expanded clones are positively selected against cancer evolution and the acquisition of additional mutations in the expanded clones results in cancer.

Previous studies have shown that the phenotypically normal “mutated” clones that exist around cancer cells could regulate the proliferation of cancer cells. Some mutations have a much higher frequency in noncancerous tissue than in cancerous tissue (2). It has been reported that the mutated clones in non-cancerous tissue promote or conversely inhibit tumor formation in a non-cell-autonomous manner (3, 4). Therefore, mutations acquired by positively selected clones in a tissue can have distinct effects on cancer development.

Cells transformed by accumulated genetic mutations proliferate and push out surrounding nontransformed cells, forming a cell mass called a focus *in vitro*. Focus formation assays utilizing this property have been widely used to identify oncogenes (5). We previously established a focus formation assay using normal mouse mammary epithelial cells (NMuMG) and identified several novel oncogenes (6, 7, 8). NMuMG cells are transformed by the introduction of a single oncogene, and they acquire features such as morphological changes, loss of contact inhibition, and tumorigenicity (5, 9). This property might be attributed to the fact that NMuMG cells already have acquired multiple genetic aberrations, including immortality. In other words, NMuMG cells can be considered to be in a “one step short of cancer” state and have properties similar to those of phenotypically normal “mutated” clones in clonal expansion.

While performing the focus formation assay using NMuMG cells, we noticed that each cell subclone established from parental NMuMG cells had different effects on the focus-forming activity of transformed cells. From this result, we speculated that some cell populations among the surrounding nontransformed cells are capable of inhibiting focus formation, and if the mechanism could be clarified, it could be used for antitumor applications. Because subclones were established from the same cell line, slight genetic or epigenetic differences could determine the level of this suppressive ability.

In this study, we established a screening system for compounds that induce the inhibitory activities of surrounding nontransformed cells on focus formation. Consequently, we identified two compounds that induce nontransformed cells to block the expansion of oncogenic foci by transformed cells.

## Results

### Screening to identify chemical compounds that inhibit oncogenic focus formation by *KRASG12D*-expressing cells only when surrounded by nontransformed cells

**Figure 1:**
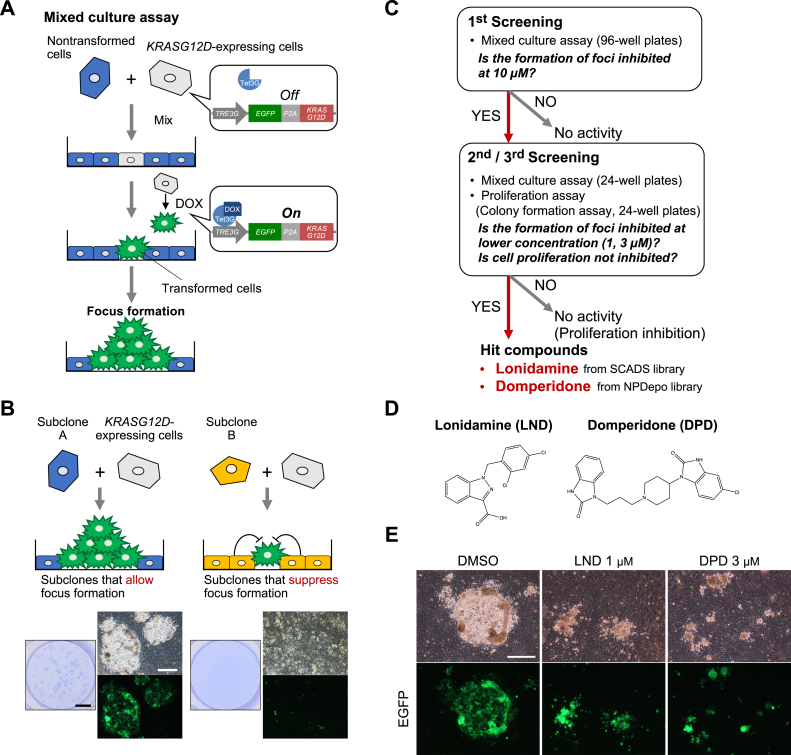
**Screening to identify chemical compounds that inhibit oncogenic focus formation by *KRASG12D*-expressing cells only when surrounded by nontransformed cells.***A*, in the established mixed culture focus formation assay, nontransformed NMuMG cells and cells expressing activated mutant *KRAS* (*KRASG12D*) by the Tet-On system were mixed (nontransformed cells:*KRASG12D*-expressing cells = 1000:1) and allowed to proliferate until reaching confluency. Then, doxycycline (DOX) was added to induce *KRASG12D* expression. After another 7 days, oncogenic foci were observed. *B*, the ability to allow or suppress focus formation differed among the subclones. Whereas most established subclones allowed *KRASG12D*-expressing cells to form oncogenic foci (illustrated as subclone A, *left*), a few subclones of surrounding nontransformed NMuMG cells efficiently suppressed focus formation by *KRASG12D*-expressing cells (illustrated as subclone B, *right*) (*left*: staining with 0.01% crystal violet, scale bar = 5 mm, *right*: phase-contrast and fluorescent images, scale bar = 500 μm). *C*, an overview of the compound screening. We screened compounds that could induce the cancer-inhibitory activities of subclone A illustrated in Fig. 1*B*. We first screened compounds exerting suppressive effects on focus formation by *KRASG12D*-expressing cells using 96-well culture plates. All compounds were used at 10 μM. From this screening, we selected candidate compounds, and their inhibitory effects were confirmed in repeated assays that were performed in lower concentrations (1 and 3 μM) using 24-well culture plates (second and third screenings). At the same time, the identified compounds were subjected to a proliferation assay to exclude the compounds that inhibited the proliferation of *KRASG12D*-expressing cells in nonixed culture. *D*, the chemical structures of lonidamine (LND) and domperidone (DPD). *E*, LND and DPD changed the morphologies and sizes of the oncogenic foci of *KRASG12D*-expressing cells at day 7 after DOX induction (*upper*: phase-contrast images; *lower*: fluorescent images, scale bar = 500 μm). NMuMG, normal murine mammary gland.

We established a mixed culture focus formation assay using NMuMG cells (Fig. 1*A*). In this assay, we mixed NMuMG cells (nontransformed cells) and NMuMG cells expressing *EGFP-2A-KRASG12D* using the Tet-ON system. When doxycycline (DOX) was added to the culture medium, a fusion protein of EGFP-2A-KRASG12D was produced and then cleaved into EGFP-2A and KRASG12D (active mutant KRAS) after translation by the ability of 2A peptide (hereafter called *KRASG12D*-expressing cells). Finally, we evaluated whether *KRASG12D*-expressing cells (transformed cells) form oncogenic foci. Although most of the subclones established from NMuMG cells allowed oncogenic focus formation by *KRASG12D*-expressing cells (Fig. 1*B*, left), a few subclones efficiently suppressed focus formation (Fig. 1*B*, right). This result suggested the existence of a mechanism by which surrounding nontransformed cells can suppress focus formation by transformed cells and that this mechanism varies among the established NMuMG subclones.

To investigate such cancer-inhibitory activities, we conducted a screening to obtain compounds that could induce the cancer-inhibitory activities of a subclone that allowed transformed cells to form oncogenic foci (subclone shown in Fig. 1*B*, left). We first screened approximately 760 compounds from the chemical libraries of the SCADS inhibitor kit and RIKEN NPDepo (Natural Product Depository) pilot library (Fig. 1*C*). The SCADS inhibitor kit consists of approximately 360 compounds. The library contains compounds such as kinase inhibitors and anticancer drugs, which have been widely used in research for molecular cell biology (http://scads.jfcr.or.jp/kit/kit.html). The NPDepo pilot library contains approximately 400 compounds representative of groups into which approximately 25,000 compounds were clustered according to their structural characteristics (10). In the first screening, we set the compound concentration to 10 μM using 96-well plates and selected candidate compounds that altered the morphology or size of the oncogenic foci. To eliminate potential candidates, the second and third screenings were performed with 24-well plates at concentrations of 1, 3, and 10 μM. Thereafter, to select compounds that specifically inhibited the formation of oncogenic foci by *KRASG12D*-expressing cells only when cultured with surrounding nontransformed cells, we excluded compounds that inhibited cell proliferation when *KRASG12D*-expressing cells were cultured alone. Finally, lonidamine (LND) from the SCADS inhibitor kit and domperidone (DPD) from the NPDepo pilot library were identified (the structures are presented in Fig. 1*D*). Under control conditions, *KRASG12D*-expressing cells formed oncogenic foci (Fig. 1*E* [dimethyl sulfoxide, DMSO], time-lapse observation of mixed culture assay, Fig. S1 [DMSO], Supplementary Movie A [DMSO]). Contrarily, LND and DPD treatment induced the oncogenic foci not to expand normally (Fig. 1*E* [LND, DPD], Fig. S1 [LND, DPD], Supplementary Movie B [LND], C [DPD]).

### LND and DPD inhibited the expansion of oncogenic foci in a cytotoxic effect-independent manner

**Figure 2:**
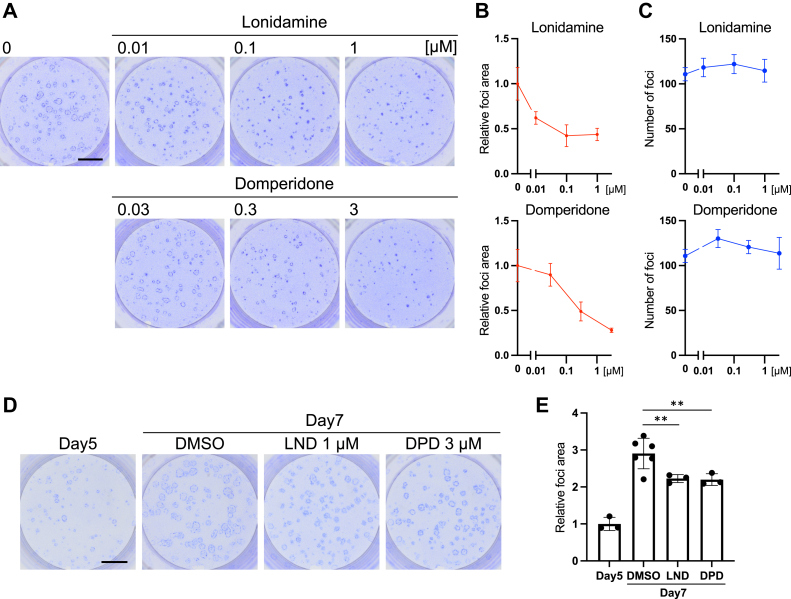
**Lonidamine (LND) and domperidone (DPD) inhibit the expansion of oncogenic foci in a concentration-dependent manner.***A*–*C*, the expansion of oncogenic foci of *KRASG12D*-expressing cells was inhibited by LND or DPD treatment. After 7 days of culture, oncogenic foci were stained with 0.01% crystal violet. Quantification of the total area (*B*) and number (*C*) of oncogenic foci based on the images of (*A*) (mean ± SD; mean and SD are obtained from measurements of six wells for DMSO and three wells for the others, scale bar = 5 mm; a representative result of two experiments is presented). The *y*-axis of (*B*) presents the ratio of the value at each concentration to that of the DMSO-treated control (0 μM). *D* and *E*, LND and DPD inhibited focus expansion even after its initiation. LND (1 μM) or DPD (3 μM) was added after foci became visible on day 5. The quantification of the total area (*E*) of oncogenic foci based on the images of (*D*) (mean ± SD; Mean and SD are obtained from measurements of six wells for DMSO and three wells for the others, ∗∗*p* < 0.01, scale bar = 5 mm; a representative result of two experiments is presented). DMSO, dimethyl sulfoxide.

Quantitative analysis revealed that the total focus area shown in Fig. 2*A* was suppressed by up to approximately 40% by 0.1 μM LND and 50% by 0.3 μM DPD compared to the control findings (Fig. 2*B*), whereas these compounds did not significantly decrease the number of foci (Fig. 2*C*). When the addition of LND or DPD was delayed until 4 days after DOX induction (day 5), at which time almost all foci had already expanded, further expansion of oncogenic foci was partly inhibited (Fig. 2, *D* and *E*). These results supported that both LND and DPD inhibited the process of focus expansion but not that of focus formation.

**Figure 3:**
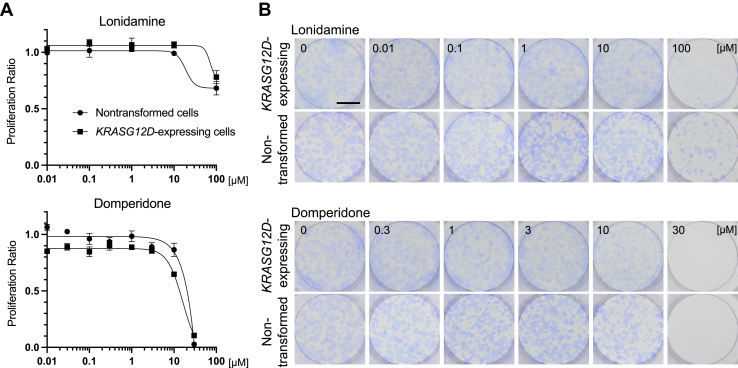
**Lonidamine (LND) and domperidone (DPD) did not inhibit cell proliferation at the concentration that inhibited focus expansion.***A*, for the short-term evaluation of cell proliferation in a nonmixed culture, *KRASG12D*-expressing cells or nontransformed cells were cultured for 48 h with/without the indicated concentration of LND or DPD, and cell viability was evaluated using Cell Counting Kit-8. The ratio of absorbance at each concentration to that at 0 μM is presented (mean ± SD; Mean and SD are obtained from measurements of three wells; a representative result of two experiments is presented). *B*, for the long-term evaluation of cell proliferation in a nonmixed culture, the colony formation assay was performed with *KRASG12D*-expressing cells or nontransformed cells with/without the indicated concentration of LND or DPD. Cells were fixed with methanol and stained using 0.01% crystal violet after incubation for 6 days (scale bar = 5 mm; a representative result of two experiments is presented).

To confirm that the inhibitory effects of LND or DPD were not attributable to direct cytotoxic effects on *KRASG12D*-expressing cells, we evaluated their effects on cell proliferation using two different time spans in nonmixed cultures (monocultures). For short-term evaluation, we conducted the WST-8 assay (for 48 h, Fig. 3*A*). For long-term evaluation, we performed a colony formation assay under experimental conditions more similar to those of the mixed culture assay (6 days, Fig. 3*B*). In both cases, LND or DPD did not inhibit the proliferation of nonmixed *KRASG12D*-expressing cells at the concentration that inhibited the expansion of foci in mixed culture. These results suggested that the inhibition of focus expansion by LND and DPD in mixed culture did not involve nonspecific cytotoxic effects.

**Figure 4:**
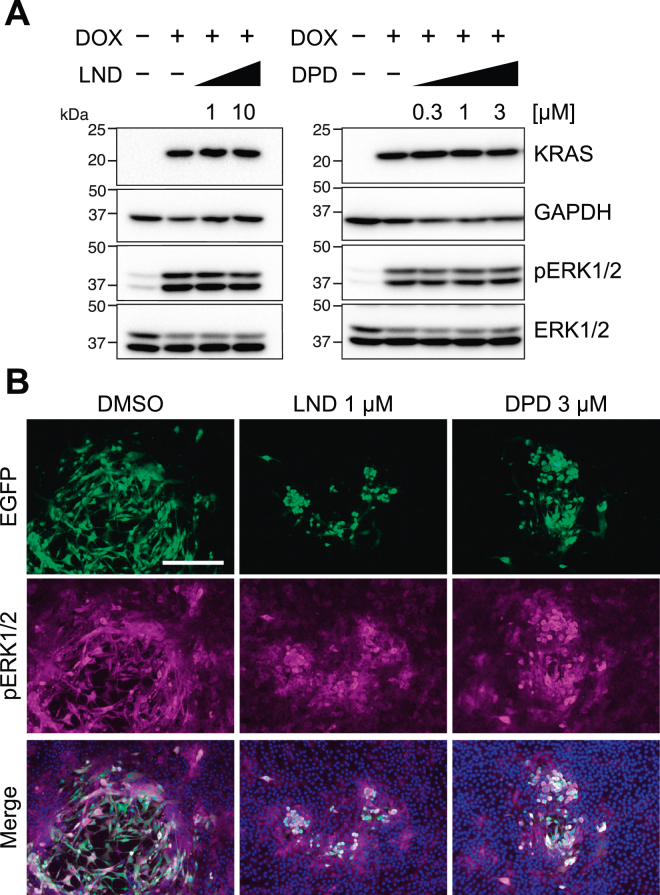
**Lonidamine (LND) and domperidone (DPD) did not inhibit KRAS signaling.***A*, LND and DPD did not inhibit KRAS expression from the vector and the phosphorylation of extracellular signal-regulated kinases 1 and 2 (ERK1/2) in a nonmixed culture. NMuMG *KRASG12D*-expressing cells were treated with 1 or 10 μM LND or 0.3, 1, or 3 μM DPD for 48 h, and KRAS expression and ERK1/2 phosphorylation were detected using Western blotting. GAPDH was used as a loading control (a representative result of four experiments is presented). *B*, LND and DPD did not inhibit ERK1/2 phosphorylation in mixed culture. The mixed culture assay was performed with treatment with 1 μM LND or 3 μM DPD for 7 days on poly-l-lysine–coated cover glass. *KRASG12D*-expressing cells expressed EGFP (*green*), the activation of the downstream pathway was detected by staining phosphorylated ERK1/2 (*magenta*), and cells were stained with DAPI (scale bar = 500 μm; a representative result of two experiments is presented). DAPI, 4′,6-diamidino-2-phenylindole; NMuMG, normal murine mammary gland.

Next, we examined whether LND and DPD inhibited KRAS signaling. We performed Western blotting using lysate harvested from *KRASG12D*-expressing cells cultured alone. KRAS expression was not reduced by 1 μM LND or 3 μM DPD treatment for 48 h (Fig. 4*A*). Furthermore, activation of extracellular signal–regulated kinases 1 and 2 (ERK1/2) downstream of the RAS-signaling pathway was not affected. Second, using the mixed culture assay, we conducted immunostaining of phospho-ERK1/2 (Fig. 4*B*). No inhibition of phospho-ERK1/2 was observed after 7 days of treatment with LND and DPD. These results indicated that LND and DPD did not inhibit KRAS signaling.

**Figure 5:**
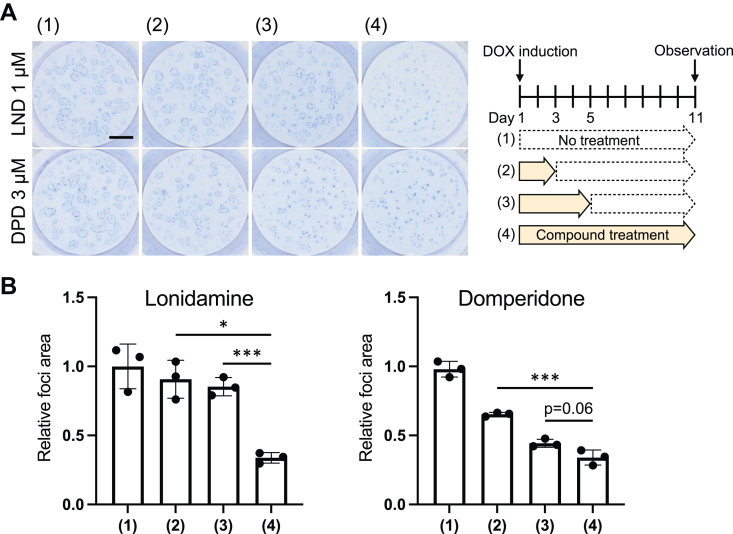
**Lonidamine (LND) and domperidone (DPD) reversibly inhibited focus expansion.***A*, the mixed culture assay was performed with/without LND (1 μM) or DPD (3 μM) treatment according to the schedule presented on the right of (*A*). The quantification of the total area of oncogenic foci (*B*) based on the images of (*A*) (mean ± SD; Mean and SD are obtained from measurements of three wells, ∗*p* < 0.05 and ∗∗∗*p* < 0.001, scale bar = 5 mm; a representative result of two experiments is presented).

To examine whether the inhibitory effects of LND and DPD were reversible, we performed LND and DPD treatment only for the first 3 or 5 days of an 11-day culture (the schedule is presented on the right of Fig. 5*A*). As a result, most of the foci started expanding after the removal of these compounds, especially under LND-treated conditions (Fig. 5, *A* and *B*). These results demonstrated the reversibility of the inhibitory effects of LND and DPD.

### Inhibitory effects of LND and DPD on focus expansion are independent of inhibition of their known targets

**Figure 6:**
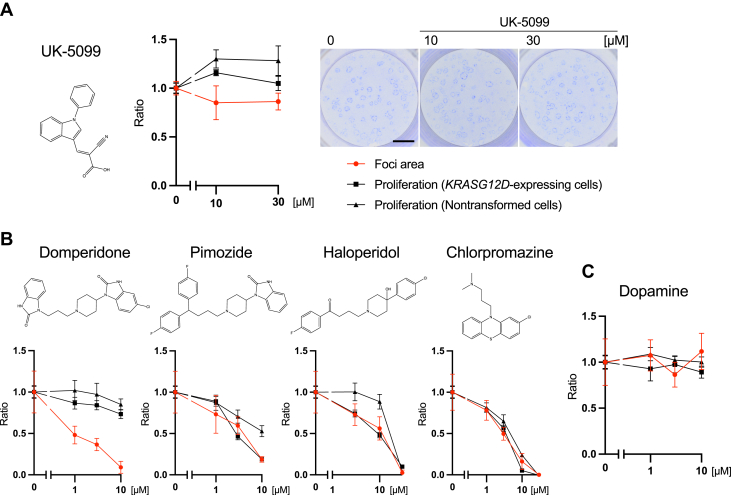
**Inhibitory effects of lonidamine and domperidone are independent of inhibition of their known targets.***A*, UK-5099, another mitochondrial pyruvate carrier inhibitor, was evaluated, but it did not inhibit focus expansion (scale bar = 5 mm, mean ± SD; Mean and SD are obtained from measurements of three wells; a representative result of two experiments is presented). *B*, other dopamine 2 receptor antagonists did not inhibit focus expansion in a cytotoxic effect–independent manner. Pimozide, haloperidol, and chlorpromazine did not inhibit focus expansion at concentrations at which cell proliferation was not inhibited (mean ± SD; Mean and SD are obtained from measurements of two wells for the focus area and three wells for cell proliferation; a representative result of two experiments is shown). *C*, dopamine had no effects on focus expansion (mean ± SD; Mean and SD are obtained from measurements of two wells for the focus area and three wells for cell proliferation; a representative result of two experiments is presented).

To examine the mechanism of action of LND and DPD, we investigated whether previously known targets of LND or DPD are involved in their inhibition of focus expansion by *KRASG12D*-expressing cells. One of the known inhibitory targets of LND is mitochondrial hexokinase 2 (11), which exists mainly in cancer cells (12). However, the widely used concentration of LND for hexokinase inhibition exceeds 100 μM. Similarly, for other several known targets (*e.g.*, mitochondrial complex II, Bcl-2, and cystic fibrosis transmembrane conductance regulator), the effective concentrations of LND are in a few 100 μM range (13, 14, 15, 16). On the contrary, LND inhibited focus expansion even at 100 nM (Fig. 2, *A* and *B*), and thus, we excluded the known targets of LND as targets of the inhibitory phenotype. Recently, LND was reported to inhibit mitochondrial pyruvate carrier (MPC) (17). MPC transports pyruvate from the cytosol into the mitochondrial matrix (18). Therefore, we used UK-5099, another MPC inhibitor (the structure is presented in Fig. 6*A*) (19). However, the inhibitory effect of UK-5099 was barely observed (Fig. 6*A*). Therefore, MPC was not suggested to be a major target of LND for its inhibitory effect on focus expansion.

DPD is a dopamine D2 receptor (D2R) antagonist. We performed the mixed culture assay using other three D2 antagonists: pimozide, haloperidol, and chlorpromazine (Fig. 6*B*). In contrast to DPD, those antagonists inhibited the expansion of oncogenic foci as well as cell proliferation. Furthermore, dopamine, the ligand for D2R, had no effects (Fig. 6*C*). These results suggested that the inhibitory effects of DPD were independent of D2R-mediated activity.

### LND and DPD inhibited cell motility at the nontransformed cell–transformed cell boundary

**Figure 7:**
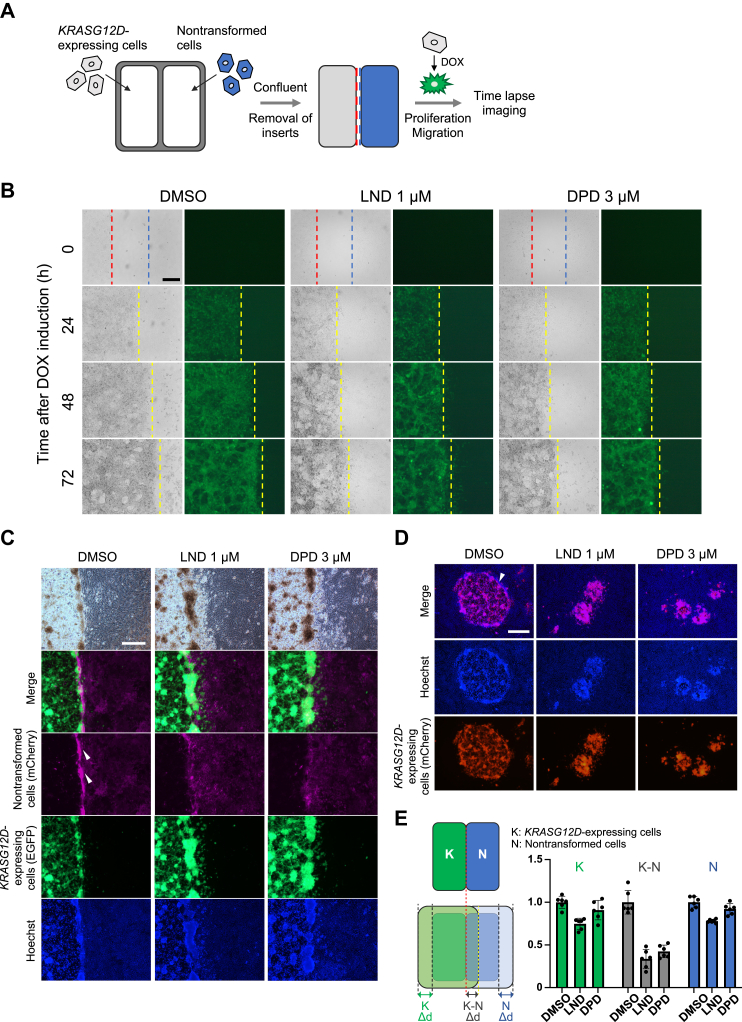
**Lonidamine (LND) and domperidone (DPD) inhibited cell motility at the nontransformed cell–transformed cell boundary.***A*, observation of the boundary between nontransformed cells and *KRASG12D*-expressing cells in the cell confrontation assay. Nontransformed cells and *KRASG12D*-expressing cells were seeded in silicone culture inserts and cultured until they became confluent. After the removal of the insert, both cells migrated and collided with each other. *B*, the spreading of *KRASG12D*-expressing cells after colliding with nontransformed cells was inhibited by LND and DPD treatment. Time-lapse images of the cell confrontation assay at the indicated times after insert removal and treatment with each compound (1 μM LND or 3 μM DPD) are presented. The first position of cells after removal of the inserts is presented as *red broken lines* for *KRASG12D*-expressing cells and as *blue broken lines* for nontransformed cells, and the boundaries between nontransformed cells and *KRASG12D*-expressing cells are presented as *yellow broken lines*. The scale bar represents 250 μm. *C* and *D*, LND and DPD inhibited the increase in the cell density of nontransformed cells caused by them being pushed by *KRASG12D*-expressing cells in the cell confrontation assay (*C*) and mixed culture assay (*D*). In the cell confrontation assay (*C*), nontransformed cells that expressed mCherry and *KRASG12D*-expressing cells that expressed EGFP were seeded into culture inserts. In the mixed culture assay (*D*), nontransformed cells (with no fluorescence labeling) and *KRASG12D*-expressing cells that expressed mCherry were used. Cells were cultured with or without the compounds for 7 days and observed by fluorescent microscopy after staining with Hoechst. The area with a high cell density of nontransformed cells is denoted by arrowheads. The scale bar represents 500 μm. *E*, LND and DPD inhibited cell motility, especially at the boundary between nontransformed cells and transformed cells. The relative migration distance (Δd) compared with DMSO control is presented. Δd of *KRASG12D*-expressing cells at the boundary between nontransformed cells and *KRASG12D*-expressing cells (K-N, *gray*) and that of *KRASG12D*-expressing cells (K, *green*) or nontransformed cells (N, *blue*) that could freely proliferate and migrate were measured by ImageJ (mean ± SD; Mean and SD are obtained from six measurements; a representative result of two experiments is presented). DMSO, dimethyl sulfoxide.

To clarify the events occurring at the boundary between nontransformed cells and *KRASG12D*-expressing cells, we seeded nontransformed cells and *KRASG12D*-expressing cells into each compartment of silicone inserts to prevent the cells from mixing and removed them after the cells became confluent (cell confrontation assay, illustrated in Fig. 7*A*). Both cells proliferated and migrated to fill the gaps and finally contacted each other. In the control experiment, *KRASG12D*-expressing cells continued to progress and expand their territory even after colliding with nontransformed cells (Fig. 7*B* [DMSO]). At the boundary between the nontransformed cells and *KRASG12D*-expressing cells, the nontransformed cells moved backward as the *KRASG12D*-expressing cells progressed (Supplementary Movie D [DMSO]). In this process, *KRASG12D*-expressing cells pushed away the nontransformed cells rather than spreading over them (Fig. 7*C*). Consequently, the density of the nontransformed cells increased (arrowheads in Fig. 7*C*). A similar tendency was also observed in the mixed culture assay (arrowhead in Fig. 7*D*). Contrarily, treatment with LND or DPD inhibited the backward movement of nontransformed cells, causing them to remain in their original position (Supplementary Movie E [LND] and F [DPD]), resulting in inhibition of the spreading of *KRASG12D*-expressing cells (Fig. 7*B* [LND] [DPD]). Because it became difficult for *KRASG12D*-expressing cells to push nontransformed cells away, the density of *KRASG12D*-expressing cells increased and some of the *KRASG12D*-expressing cells at the frontline appeared to ride over the layer of nontransformed cells (Fig. 7*C* [LND] [DPD]). Quantitative analysis of migration distances illustrated that LND and DPD inhibited the spreading of *KRASG12D*-expressing cells at the cell boundary by approximately 60% to 70% (Fig. 7*E* [K-N], *gray*). On the other hand, the spreading of *KRASG12D*-expressing cells (Fig. 7*E* [K], *green*), as well as nontransformed cells (Fig. 7*E*[N], *blue*), toward free space was inhibited by only about 10% to 30% by LND or DPD treatment. Therefore, the effects of LND and DPD on cell motility were specific to the region of the cell boundary. These results demonstrated that LND and DPD inhibited focus expansion by markedly inhibiting cell motility of nontransformed cells at the boundary between nontransformed and *KRASG12D*-expressing cells.

### Enhanced actin stress fiber formation in surrounding nontransformed cells might affect the ability of transformed cells to form foci

**Figure 8:**
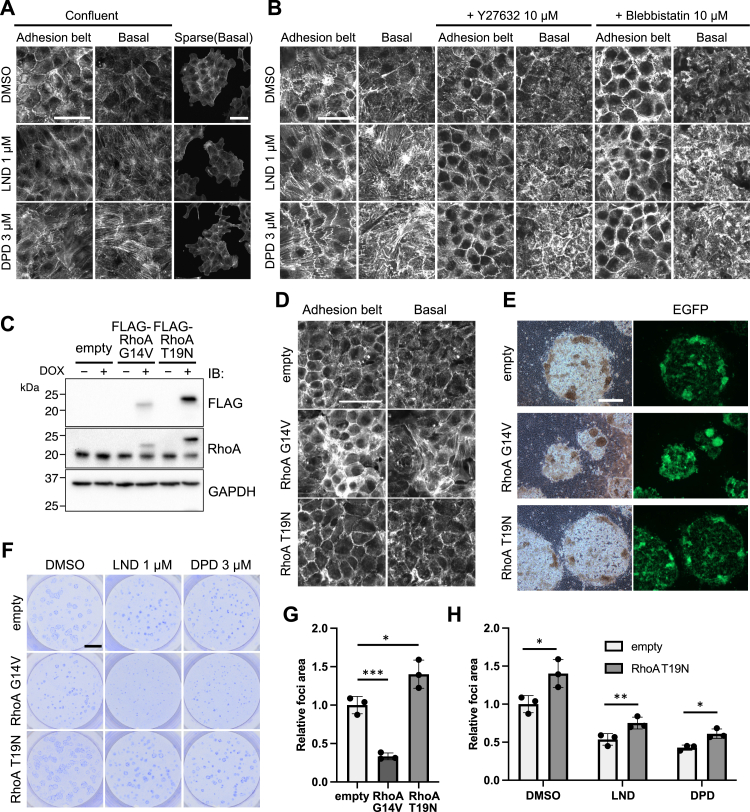
**Enhanced actin stress fiber formation in surrounding nontransformed cells might affect the ability of transformed cells to form foci.***A*, the localization of F-actin at the adhesion belt and basal planes was altered by lonidamine (LND, 1 μM) or domperidone (DPD, 3 μM) treatment (*left*), whereas sparse cells did not form stress fibers (*right*). Thus, a confluent condition is required for the enhancement of actin stress fiber formation by LND and DPD. Nontransformed cells were seeded on glass coverslips coated with poly-l-lysine in 12-well plates at a density of 2.0 × 10^5^ (confluent) or 2.0 × 10^4^ cells/well (sparse). Cells were treated with LND or DPD for 48 h and then stained with phalloidin. The scale bar represents 50 μm. *B*, treatment with the Rho-associated kinase inhibitor Y27632 (10 μM) or myosin II inhibitor blebbistatin (10 μM) abrogated the enhancement of actin stress fiber formation by LND or DPD. Nontransformed cells were pretreated with Y-27632 or blebbistatin for 1 h, and after 48 h of incubation with LND (1 μM) or DPD (3 μM), cells were stained with phalloidin. The scale bar represents 50 μm. *C*, the expression of FLAG-RhoA (constitutive active [CA] or dominant-negative [DN]) protein in Tet3G-expressing nontransformed NMuMG cells was examined using Western blotting. *D*, stress fiber formation of RhoA mutant-expressing nontransformed cells. Nontransformed cells were seeded on glass coverslips coated with poly-L-lysine in 12-well plates at a density of 2.0 × 10^5^ cells/well. DOX was added to the culture media 2 days later and cells were cultured for 24 h and then stained with phalloidin. The scale bar represents 50 μm. *E*–*G*, CA or DN RhoA mutant-expressing nontransformed cells affected focus expansion by *KRASG12D*-expressing cells (*E*: phase-contrast and fluorescent images, the scale bar represents 500 μm; *F*: staining with 0.01% crystal violet, the scale bar represents 5 mm). *G*, quantification of the total area of oncogenic foci was based on the images of (*F*) (mean ± SD; Mean and SD are obtained from measurements of three wells, ∗*p* < 0.05, ∗∗∗*p* < 0.001; a representative result of three experiments is presented). *H*, the inhibitory effects of LND (1 μM) and DPD (3 μM) were partly canceled in the mixed culture assay using DN RhoA-expressing nontransformed cells. Quantification of the total area of oncogenic foci was based on the images of (*F*) and relative focus area compared to the empty vector and DMSO control is presented (mean ± SD; Mean and SD are obtained from measurements of three wells, ∗*p* < 0.05, ∗∗*p* < 0.01; a representative result of three experiments is presented). DMSO, dimethyl sulfoxide, DOX, doxycycline; NMuMG, normal murine mammary gland.

The observation of the effects on cell motility led us to analyze the effects on actin fibers by LND or DPD. We first stained F-actin using phalloidin. When nontransformed cells were treated with LND or DPD, enhanced actin stress fiber formation was observed over most basal planes of the cells (Fig. 8*A*, left). In addition, this enhanced actin stress fiber formation was not observed when cells were seeded sparsely (Fig. 8*A*, right). The formation of actin stress fibers was abrogated by treatment with the Rho-associated coiled-coil containing protein kinase (ROCK) inhibitor Y27632 and myosin II inhibitor blebbistatin (Fig. 8*B*), suggesting that the stimulating effects of LND and DPD on actin stress fiber formation are regulated by ROCK and myosin II pathways.

RhoA is a Rho family GTPase that regulates cell motility and induces myosin II–dependent contraction through ROCK activation, which results in stress fiber formation (20, 21). These observations led us to hypothesize that LND or DPD treatment enhances RhoA activity in nontransformed cells and inhibits focus expansion. To investigate the effects of RhoA activity in nontransformed cells on the expansion of oncogenic foci, we introduced a constitutive active (CA) mutant of RhoA (*RhoA G14V*) or dominant-negative (DN) mutant of RhoA (*RhoA T19N*) (22, 23) into Tet3G-expressing nontransformed cells. The inducible expression of each FLAG-RhoA protein and actin stress fiber formation was confirmed in Fig. 8, *C* and *D*, respectively. Using these RhoA-expressing nontransformed cells, we performed the mixed culture assay. Nontransformed cells expressing CA RhoA prevented focus expansion by *KRASG12D*-expressing cells (Fig. 8, *E*–*G*). The expression of a different CA mutant, namely *RhoA Q63L*, also inhibited focus expansion (Fig. S2, *A*–*E*). Conversely, nontransformed cells expressing DN RhoA allowed *KRASG12D*-expressing cells to form larger foci (Fig. 8, *E*–*G*). The inhibitory effects of LND or DPD were partly canceled by the induction of DN RhoA expression (Fig. 8*H*).

In addition to RhoA, Rac1 also plays an important role in cell motility (24, 25, 26). Therefore, we performed the mixed culture assay using a DN mutant of Rac1 (*Rac1 T17N*) (27). Nontransformed cells expressing DN Rac1 (the expression of each FLAG-Rac1 protein was confirmed in Fig. S2*F*) allowed *KRASG12D*-expressing cells to form larger foci (Fig. S2, *G*–*I*). The inhibitory effects of LND or DPD were partly canceled by the induction of DN Rac1 expression (Fig. S2*I*). These results suggested that altered regulation of cell motility in nontransformed cells could affect focus expansion by *KRASG12D*-expressing cells. In addition, consistent with a previous report (28), cells expressing a CA mutant of Rac1 (*Rac1 G12V*) were morphologically altered; thus, the mixed culture assay could not be performed.

We investigated whether the RhoA/Rac1 regulatory pathway is involved in blocking the focus expansion of a subclone (shown on the right in Fig. 1*B*; hereafter called subclone B). Subclone B showed enhanced stress fiber formation (Fig. S3*A*) compared with subclones that allowed focus expansion (shown on the left in Fig. 1*B*). Next, we introduced the expression of DN mutants of RhoA (RhoA T19N) or Rac1 (Rac1 T17N) in Tet3G-expressing subclone B (Fig. S3, *B* and *C*) and performed the mixed culture assay. DN Rac1-expressing cells allowed *KRASG12D*-expressing cells to form larger foci (Fig. S3, *D* and *E* [Rac1 T17N]), whereas DN RhoA expression did not affect focus formation substantially (Fig. S3, *D* and *E* [RhoA T19N]), which may be due to the relatively low expression of DN RhoA (Fig. S3*B*). These results suggested that the RhoA/Rac1 regulatory pathway partially inhibited focus expansion even of subclone B.

## Discussion

The proliferation of transformed cells is suppressed by the surrounding nonransformed cells, such as primary fibroblasts or keratinocytes (29, 30, 31, 32, 33). In epithelial cancer, cells that have acquired mutations were reported to be physically eliminated by the surrounding nontransformed cells for epithelial defense (34). However, when these anticancer mechanisms mediated by the surrounding nontransformed cells fail to function properly, cells with mutations remain in the nontransformed cell layer and precancerous lesions are formed (35). With aging, clonal expansion occurs in many noncancerous human tissues, and a patchwork of mutant clones that have accumulated somatic mutations is formed. NMuMG cells accumulate mutations and are in a “one step short of cancer” state. One additional step, such as the acquisition of Ras mutation, can fully transform them. We think that our assay system *in vitro* using NMuMG cells mimics clonal expansion and emergence of transformed cells *in vivo*.

**Figure 9:**
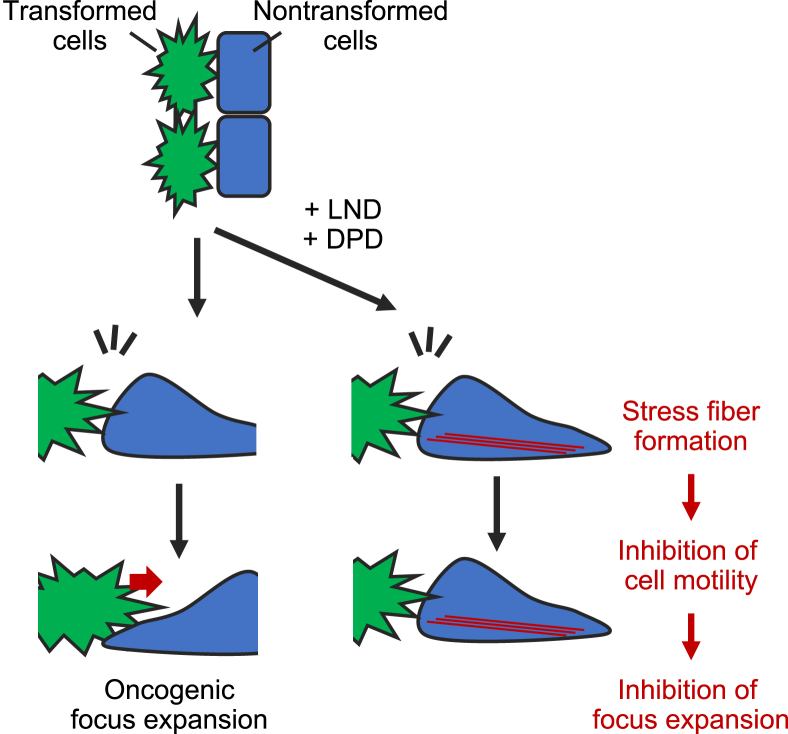
**Hypothetical schematic model of the inhibition of oncogenic focus expansion by lonidamine (LND) and domperidone (DPD).** LND and DPD inhibited cell motility at the boundary between nontransformed cells and transformed cells by enhancing actin stress fiber formation in nontransformed cells, resulting in the suppression of focus expansion by transformed cells.

The acquired compounds, LND and DPD, inhibited the cell motility of nontransformed cells moving backward as *KRASG12D*-expressing cells progressed. As a result, the movement of *KRASG12D*-expressing cells to expand their own “territory” by pushing out nontransformed cells was suppressed (Fig. 9). We also found that LND and DPD promoted actin stress fiber formation through activation of the ROCK–myosin II pathway. Indeed, the introduction of CA RhoA, which is known to regulate processes upstream of the ROCK–myosin II pathway, suppressed focus expansion, whereas the introduction of the DN mutant, which partially inhibited the signaling pathway, promoted focus expansion. These results suggested that enhanced stress fiber formation by LND or DPD resulted in the suppression of cell motility, and this effect was at least partially regulated by RhoA. In addition, the introduction of a DN mutant of Rac1, which is responsible for the regulation of cell motility by spatiotemporally switching activity coupled with RhoA, also promoted focus expansion. This result also supported the model that inhibition of the backward movement of nontransformed cells led to the inhibition of focus expansion. The mechanism by which the compounds regulate the activity of those G-proteins remains to be examined. In addition, it should be noted that the suppression of LND and DPD activity by DN mutants of RhoA and Rac1 was partial. This result suggested that LND and DPD exerted their function in a RhoA- and Rac1-independent manner. To reveal the molecular mechanism underlying the function of LND and DPD, analysis of LND- and DPD-binding proteins is in progress.

LND, first identified as an antispermatogenic agent (36), is a derivative of indole-3-carboxylic acid and has limited anticancer activity as a single agent. However, LND improves the efficacy of conventional therapies, such as chemotherapy and radiation therapy (37). Given the significant differences between the concentrations at which inhibition of known targets has been observed *in vitro* (a few 100 μM) and those used in this study (low micromolar), the known targets of LND are not likely to be involved in its inhibitory effects. In addition, UK-5099 did not inhibit focus expansion at 10 μM even though UK-5099 inhibits MPC more potently than a-cyano-4-hydroxycinnamate (19), which inhibits MPC as potently as LND (17). These results suggested that MPC was not the target that related to the inhibitory effects of LND.

DPD is a benzimidazole compound that acts as a peripheral D2R antagonist because it hardly crosses the blood–brain barrier. It is structurally related to haloperidol and other butyrophenone tranquilizers (38). Because increased dopamine receptor expression is observed in many cancers, some reports have focused on the anticancer activity of D2R antagonists other than DPD (39). However, it has also been reported that the concentration required for the cytotoxic effects of D2R antagonists exceeds their affinity for the receptors, and these compounds may have non-D2R targets (40). We demonstrated that inhibitory effects for focus expansion were not observed for other D2R antagonists and focus expansion was not regulated by dopamine. These results suggested an unknown target of DPD rather than D2R was responsible for the inhibitory effects. Taken together, we revealed new functions of known compounds that have been used to treat other diseases and target other receptors and demonstrated the potential for repositioning existing drugs.

Contact inhibition of locomotion (CIL) is a phenomenon related to cell motility that occurs when cells encounter each other. CIL has been identified in many cells during development, including embryonic fibroblasts, neural crest cells, and hematopoietic cells. CIL consists of four major steps: cell–cell contact, inhibition of cell protrusion at the contact site, formation of new protrusions and repolarization of cells, and movement away from the collision site, which overlap and progress continuously. The changes in the direction of movement are necessary for the process to complete (41). Switching the activation of RhoA and Rac1 is important for changes in the direction, and it has been reported that inhibition or the use of CA mutants of RhoA and Rac1 inhibited CIL (42). It is also known that CIL is lost between cancer cells and noncancerous cells. A model has also been proposed in which cancer cells, after colliding with noncancerous cells, continue to move on top of noncancerous cells without changing their direction of movement (43, 44). In contrast, the localization of nontransformed cells (mCherry) and *KRASG12D*-expressing cells (EGFP) did not merge in our experiments; therefore, that model differs from our finding that nontransformed cells moved backward upon collision. Our observation is consistent with a previous report of the regression of nontransformed cells against immortalized human embryonic kidney cells expressing RAS (45). Aberrant CIL is commonly observed among cancer cells; however, the behavior of cancer cells at the border between noncancerous and cancer cells may depend on the cancer cell type or a combination of the two cell types.

Anticancer drugs that target noncancerous cells surrounding cancer cells have been studied in recent years. Because of their genome stability, these anticancer agents are expected to evade drug resistance and exhibit a different mechanism of action from conventional agents that target the proliferation of cancer cells. Therefore, therapeutic strategies that combine both activities are also anticipated. Similar in concept to our study, a prior study screened compounds that do not inhibit proliferation when cancer-associated fibroblasts or cancer cells are cultured alone but inhibit proliferation when cancer-associated fibroblasts and cancer cells are cocultured (46). However, the compound identified by Kawada *et al.* targets the proliferation mechanism mediated by humoral factors between cancer-associated fibroblasts and cancer cells, and it is believed to have a different mechanism of action than the compounds identified in this study that target cell motility. *In vivo* assessment of LND and DPD using a suitable tumor model remains to be performed.

In conclusion, we have established a screening system and identified compounds that regulated the oncogenic focus expansion of transformed cells by regulating the cell motility of nontransformed cells surrounding the transformed cells. Analysis of the mechanism of action of these compounds and evaluation of their anticancer effects could lead to a new therapeutic strategy for cancer.

## Experimental procedures

### Cell culture

NMuMG cells were cultured in low-glucose Dulbecco’s modified Eagle’s medium (DMEM) containing l-glutamine and phenol red (041-29775, FUJIFILM Wako Pure Chemical Corporation) supplemented with 10% (v/v) fetal bovine serum, 10 μg/ml insulin (096-03443, FUJIFILM Wako Pure Chemical Corporation), d-glucose (final concentration, 4500 mg/L; 041-00595, FUJIFILM Wako Pure Chemical Corporation), 100 U/ml penicillin (Meiji-Seika Pharma Co, Ltd), and 100 μg/ml streptomycin (Meiji-Seika Pharma) at 37 °C in an atmosphere of 5% CO_2_. NMuMG cells were subjected to a limiting dilution method, and the isolated clones were used in this research. Plat-E packaging cells were cultured in low-glucose DMEM containing 10% (v/v) fetal bovine serum, 100 U/ml penicillin, and 100 μg/ml streptomycin at 37 °C in a 5% CO_2_ atmosphere.

### Gene expression

To generate *KRASG12D*-expressing cells, Tet3G-expressing NMuMG cells were transfected with pPB-*TRE3G-EGFP-P2A-KRASG12D*-pUbC-*Puro*^*R*^ and the hyperactive *piggyBac* transposase (hyPBase) expression vector (pCMV-*hyPBase*) (47). The cells were selected in bulk with puromycin (2 μg/ml) and subcloned. The expression of *mCherry* was induced depending on the purpose of the experiments. Full-length mouse *RhoA* and *Rac1* complementary DNA was obtained from NMuMG complementary DNA and subcloned into the pPB vector, and the subcloning was confirmed by sequencing. Tet3G-expressing NMuMG cells were transfected with pPB-*TRE3G-FLAG-RhoAT19N*-pUbC-*Puro*^*R*^, pPB-*TRE3G-FLAG-RhoAG14V*-pUbC-*Puro*^*R*^, pPB-*TRE3G-FLAG-RhoAQ63L*-pUbC-*Puro*^*R*^, pPB-*TRE3G-FLAG-Rac1T17N*-pUbC-*Puro*^*R*^, and *hyPBase* expression vectors. pPB-*TRE3G-FLAG*-pUbC-*Puro*^*R*^ vectors served as the negative control. The cells were selected in bulk with puromycin (2 μg/ml). Transfection of these vectors was performed using Opti-MEM (31985-070) and PEI (#24765-2).

### Chemical reagents

DPD (18875), pimozide (16222), LND (14640), and UK-5099 (16980) were purchased from Cayman Chemical. Haloperidol (084-04261), 3,4-dihydroxyphenethylamine hydrochloride, and dopamine hydrochloride (040-15433) were purchased from FUJIFILM Wako Pure Chemical Corporation. Chlorpromazine (C2481) was purchased from Tokyo Chemical Industry Co, Ltd. Y27632 (Y-5301) was purchased from LC Laboratories. (S)-(−)-blebbistatin (B592500) was purchased from Toronto Research Chemicals. For cell culture experiments, all chemical reagents were dissolved in DMSO (FUJIFILM Wako Pure Chemical Corporation) for the stock solution and stored at −20 °C.

### Mixed culture focus formation assay

For the mixed culture focus formation assay (mixed culture assay), 2.0 × 10^5^ NMuMG or NMuMG-mCherry cells (cells infected with retroviral particles using pMXs-*mCherry-IRES-Puro*^*R*^) and 200 *KRASG12D*-expressing cells were seeded into 12-well culture plates. After cells formed a confluent cell layer, the culture medium was replaced with fresh medium containing 50 ng/ml DOX with vehicle (DMSO) or each compound at the indicated concentrations, and the medium was subsequently replaced every other day. After incubation for 7 days, phase-contrast and fluorescent images of cells were taken, and cells were fixed with methanol and stained using 0.01% crystal violet. Quantification of the area of foci was performed using Open CV (48). Cells were stained with Hoechst 33342 (346-07951, Dojindo). For live imaging, cells were observed using CytoWatcher FL microscopes (WSL-1800-B, ATTO CORPORATION) every 10 min.

### Cell confrontation assay

Nontransformed NMuMG cells and *KRASG12D*-expressing cells (3.0 × 10^4^ cells) were seeded with silicone culture inserts (Ibidi). After cells formed a confluent layer, the culture inserts were removed, and cell migration and collision were observed *via* two CytoWatcher FL microscopes every 10 min for live imaging. Cells were stained with Hoechst 33342.

### Cell proliferation assay

Cell proliferation was detected using Cell Counting Kit-8 (Dojindo) and the colony formation assay. For the Cell Counting Kit-8 assay, cells were seeded into 96-well plates at a density of 1000 cells per well. The following day, the culture medium was replaced with fresh medium containing 50 ng/ml DOX with vehicle or each compound at the indicated concentrations. Cell viability was evaluated after 48 h of treatment using TriStar2S LB942 microplates (Berthold Technologies GmbH & Co KG) according to the manufacturer’s instructions. For the colony formation assay, cells were seeded into 24-well plates at a density of 1000 cells per well. The following day, the culture medium was replaced with fresh medium containing 50 ng/ml DOX with vehicle or each compound at the indicated concentrations. After incubation for 6 days, cells were fixed with methanol and stained using 0.01% crystal violet.

### Western blotting

Cells were harvested and lysed in TNE buffer (20 mM Tris–HCl, pH 8.0, 150 mM NaCl, 2 mM EDTA, 1% NP-40) containing phosphatase inhibitors (10 mM NaF, 1.5 mM Na_3_VO_4_) and a protease inhibitor (1 mM PMSF) on ice. The protein concentration was determined using a Pierce BCA Protein Assay Kit (Thermo Fisher Scientific). Equal amounts of protein (15 μg protein) were separated by 12.5% SDS-PAGE and transferred by electrophoresis onto polyvinylidene difluoride membranes (Immobilon-P, Millipore). Membranes were incubated overnight at 4 °C with the indicated antibodies diluted to 1:4000 (anti-K-Ras [Santa Cruz, #sc-30], anti-phospho-p44/42 MAPK [Erk1/2]) (Cell Signaling Technology, #4370), anti-p44/42 MAPK [Erk1/2] (Cell Signaling Technology, #4695), anti-DYKDDDDK (FUJIFILM Wako Pure Chemical Corporation, 014-22383), anti-RhoA (Santa Cruz, #sc-418), anti-Rac1 [BD Transduction Laboratories, 610650, and anti-GAPDH (Proteintech, 60004-1-Ig]). After three washes with Tris-buffered saline containing 0.05% Tween 20, membranes were incubated with horseradish peroxidase (HRP)conjugated secondary antibodies (antimouse IgG HRP-linked antibody [Cell Signaling Technology, #7076], anti-rabbit IgG HRP-linked antibody [Cell Signaling Technology, #7074]), and chemiluminescence was detected using Chemidoc (Bio-Rad).

### Immunofluorescent staining

Cells were seeded on glass coverslips coated with poly-l-lysine in 12-well plates and incubated for 2 days until they reached confluence. The culture medium was replaced with fresh medium containing the vehicle or each compound. After incubation for an appropriate period for the purpose of the experiment, cells were fixed with 3.7% formaldehyde/PBS for 10 min and permeabilized with 0.5% Triton X-100/PBS for 10 min. Fixed cells were blocked in 0.3% Triton X-100/PBS containing 5% goat serum for 1 h at room temperature (RT) and incubated overnight at 4 °C with primary antibodies (phospho-p44/42 MAPK) diluted in PBS containing 1% bovine serum albumin and 0.3% Triton X-100. The following day, the cells were rinsed three times with 0.1% Triton X-100/PBS for 5 min each. Proteins were labeled with secondary antibodies (goat anti-rabbit IgG [H + L] crossadsorbed secondary antibody, Alexa Fluor 568, A-11011, Invitrogen) diluted at 1:1000 and 4′,6-diamidino-2-phenylindole for 1 h at RT. For phalloidin staining, after blocking with goat serum, cells were incubated with Alexa Fluor 568 phalloidin (A12380, Invitrogen) diluted at 1:1000 and 4′,6-diamidino-2-phenylindole for 1 h at RT. After incubation, the cells were rinsed three times with 0.1% Triton X-100/PBS for 5 min each and mounted with 0.1 M Tris–HCl (pH 8.5) containing 25% glycerol, 10% MOWIOL 4-88, and 2% *n*-propyl gallate. The cells were imaged using fluorescence microscopy (Olympus Corporation).

### Statistical analysis

All statistical analyses were performed using GraphPad Prism 9 (GraphPad Software). Statistical results are presented as the mean ± SD. Welch’s *t* test was employed for comparisons between two groups.

## Data availability

All relevant data are within the paper and Supporting Information files and available upon requests.

## Supporting information

This article contains supporting information.

## Conflict of interest

The authors declare that they have no conflicts of interest with the contents of this article.

## Supporting information

Supplementary Figures and Movies

Supplementary SupMovies
