# Supplementary material for: Lonidamine and domperidone inhibit expansion of transformed cell areas by modulating motility of surrounding nontransformed cells
Source: J Biol Chem. 2022 Oct 21;298(12):102635. doi: 10.1016/j.jbc.2022.102635 (PMC9706533; doi:10.1016/j.jbc.2022.102635)
Supplement: Supplementary Figures and Movies [file mmc1.pdf]

Supporting information

Lonidamine and domperidone inhibit expansion of transformed cell areas by modulating motility of surrounding non-transformed cells

Megumi Aoyama[a*], Kosuke Ishikawa[b], Shuntaro Nemoto[a], Hiroyuki Hirano[c], Nobumoto Watanabe[c], Hiroyuki Osada [c, d], Shinya Watanabe[e], Kentaro Semba [a, e *]

[a]Department of Life Science and Medical Bioscience, School of Advanced Science and Engineering, Waseda University, 2-2 Wakamatsu-cho, Shinjuku-ku, Tokyo 162-8480, Japan
[b]Japan Biological Informatics Consortium (JBiC), 2-45 Aomi, Koto-ku, Tokyo 135-8073, Japan
[c]RIKEN Center for Sustainable Resource Science, 2-1 Hirosawa, Wako, Saitama, 351-0198, Japan
[d]Department of Pharmaceutical Sciences, University of Shizuoka, 52-1 Yada, Suruga-ku, Shizuoka 422-8526, Japan
[e]Translational Research Center, Fukushima Medical University, 1 Hikarigaoka, Fukushima 960-1295, Japan

Figures S1-S3
Supplementary Movies A-F

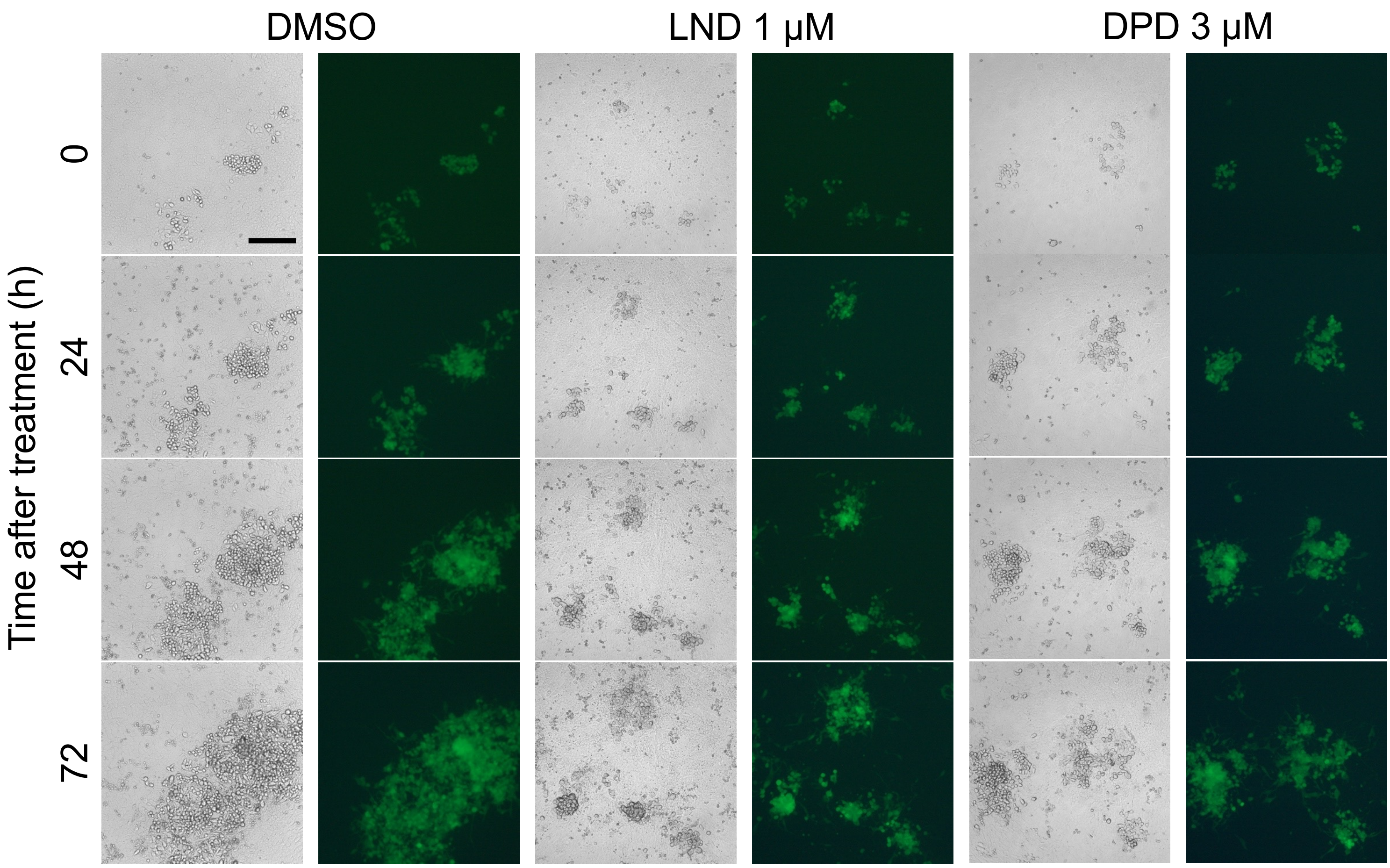

Fig. S1
Lonidamine (LND) and domperidone (DPD) changed the process of oncogenic focus formation by *KRASG12D*-expressing cells. Time-lapse images of mixed culture at the indicated times after doxycycline induction and each compound treatment (1 μM LND or 3 μM DPD). Scale bar = 250 μm.

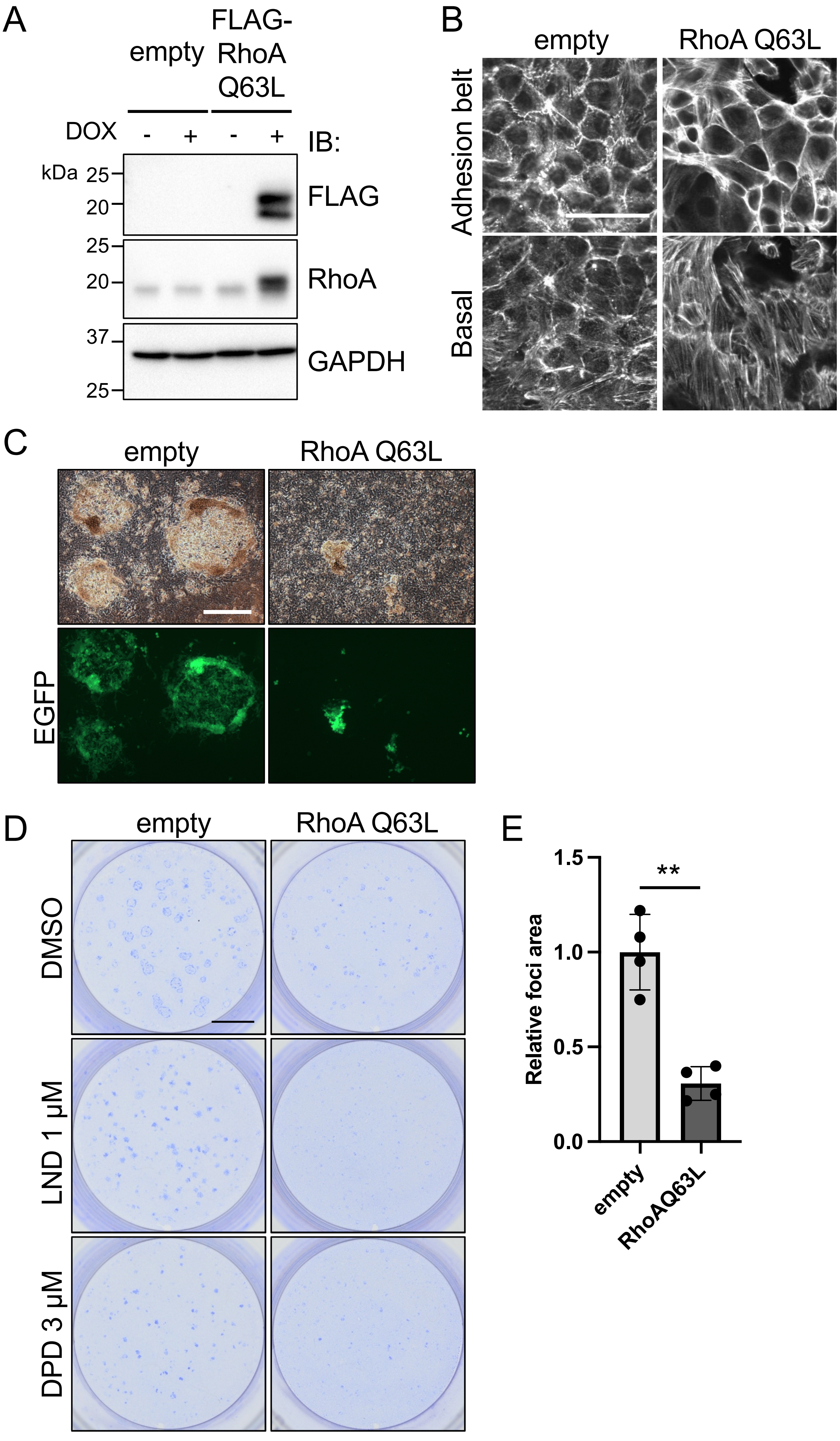
A
FLAG-
empty RhoA
Q63L
DOX - + - +
IB:
kDa 25
20
FLAG
25
20
RhoA
37
25
GAPDH
B
empty
RhoA Q63L
Adhesion belt
Basal
C
empty
RhoA Q63L
EGFP
D
empty
RhoA Q63L
DMSO
LND 1 μM
DPD 3 μM
E
1.5
1.0
0.5
0.0
**
Relative foci area
empty
RhoAQ63L

Fig.S2

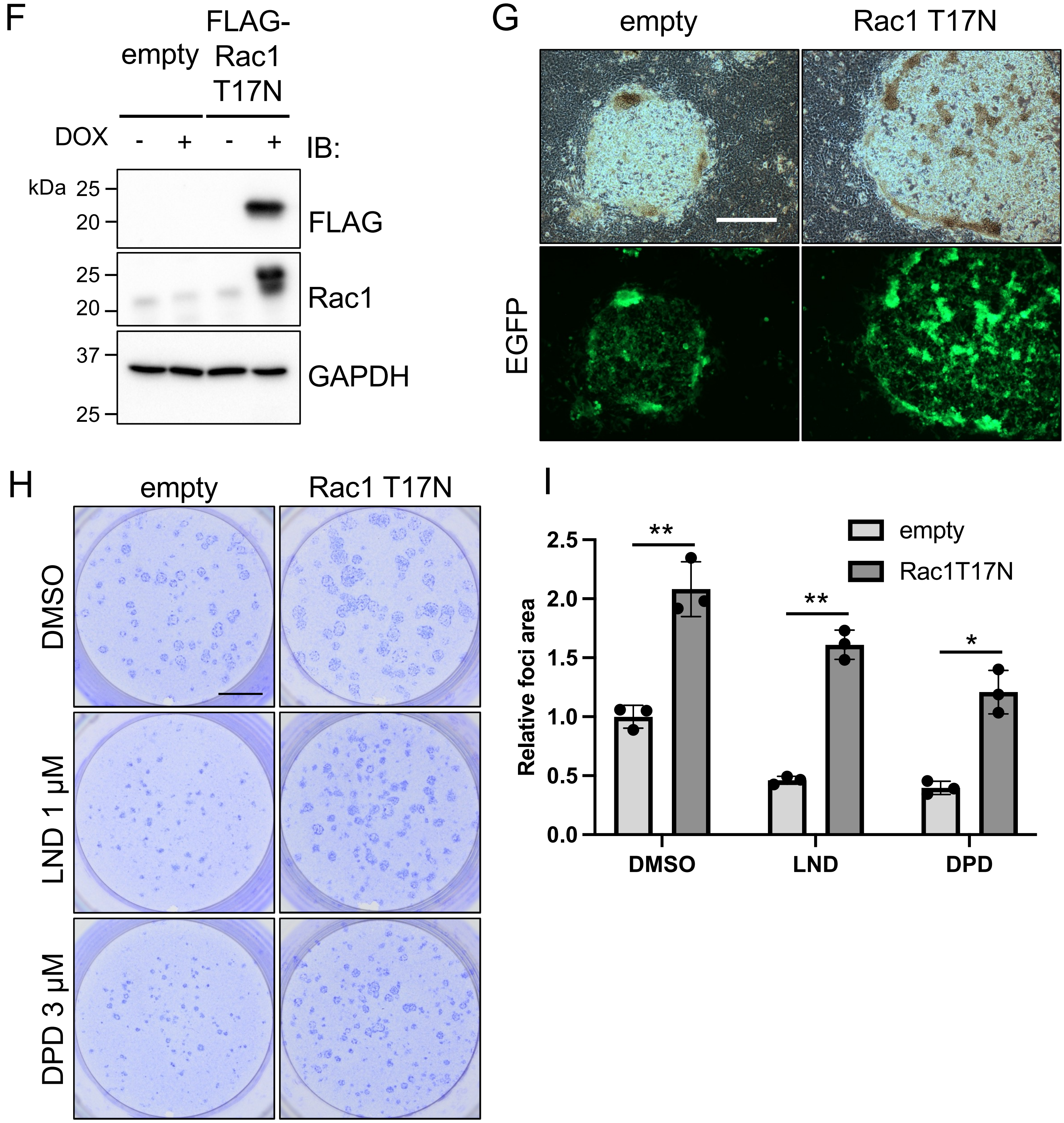
F
empty
FLAG-Rac1 T17N
DOX
-
+
-
+
IB:
kDa
25
20
FLAG
25
20
Rac1
37
25
GAPDH
G
empty
Rac1 T17N
EGFP
H
empty
Rac1 T17N
DMSO
LND 1 μM
DPD 3 μM
I
empty
Rac1T17N
Relative foci area
2.5
2.0
1.5
1.0
0.5
0.0
**
**
*
DMSO
LND
DPD

Fig.S2

Fig. S2

(A) The expression of FLAG-RhoA Q63L protein in Tet3G-expressing non-transformed NMuMG cells was examined using western blotting.

(B) RhoA Q63L mutant-expressing non-transformed cells showed enhanced stress fiber formation. Non-transformed cells were seeded on glass coverslips coated with poly-L-lysine in 12-well plates at a density of 2.0×10^5 cells per well. DOX was added to culture media two days later and cells were cultured for 24 h and then stained with phalloidin. Scale bar = 50 µm.

(C–E) RhoA Q63L mutant-expressing non-transformed cells affected focus expansion by *KRASG12D*-expressing cells (B: phase-contrast and fluorescent images, scale bar = 500 µm; C: staining with 0.01% crystal violet, scale bar = 5 mm). (D) Quantification of the total area of oncogenic foci was based on the images of (C) (mean ± SD; Mean and SD are obtained from measurements of four wells, $^{**}p < 0.01$; a representative result of three experiments is presented).

(F) The expression of dominant-negative (DN) FLAG-Rac1 (Rac1T17N) protein in Tet3G-expressing non-transformed NMuMG cells was examined using western blotting.

(G–I) DN Rac1-expressing non-transformed cells affected focus expansion by *KRASG12D*-expressing cells (G: phase-contrast and fluorescent images, scale bar = 500 µm; H: staining with 0.01% crystal violet, scale bar =5 mm). (I) Quantification of the total area of oncogenic foci was based on the images of (H). The inhibitory effects of LND or DPD were partly canceled in the mixed culture assay using DN Rac1-expressing non-transformed cells (mean ± SD; Mean and SD are obtained from measurements of three wells, $^{*}p < 0.05$, $^{**}p < 0.01$; a representative result of three experiments is presented).

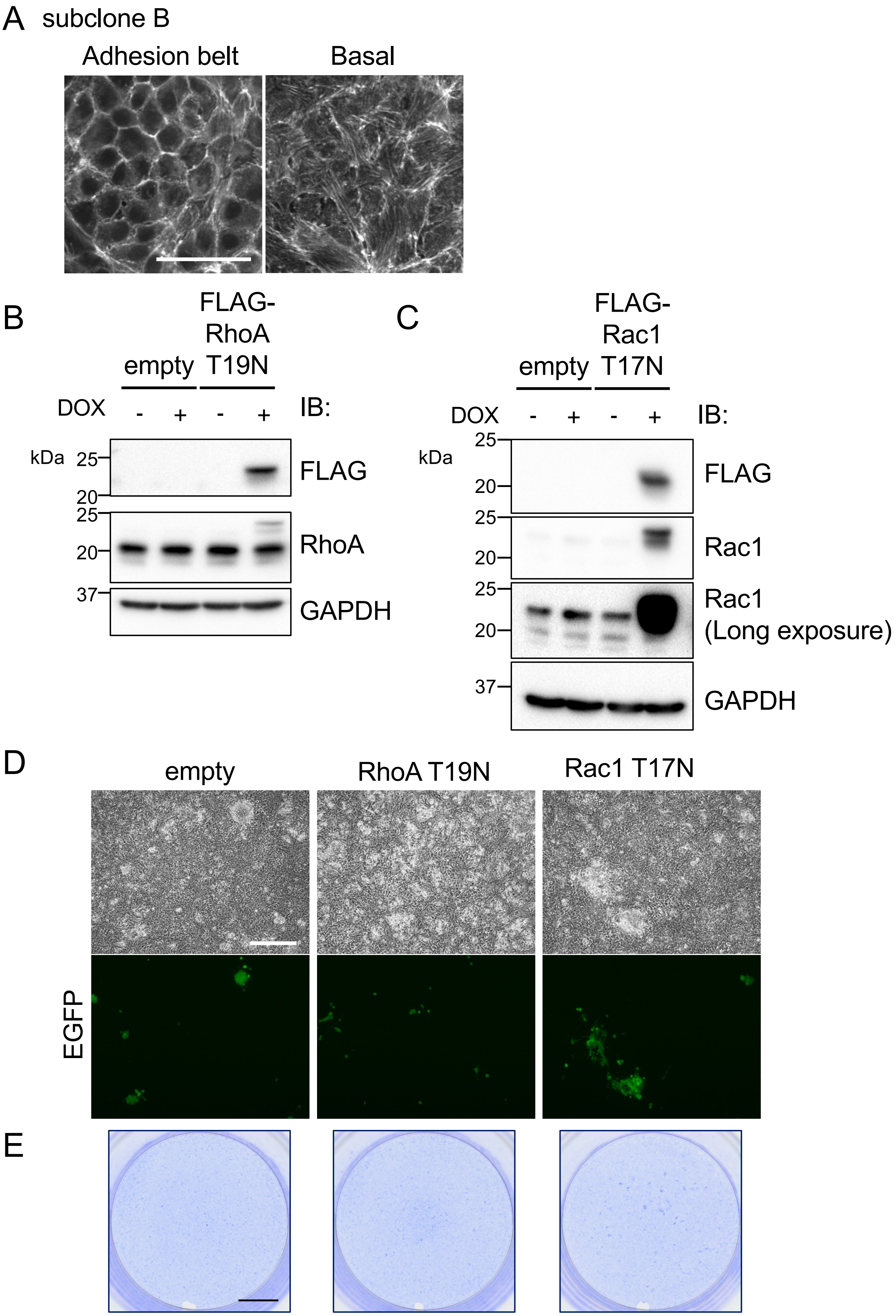
A subclone B
Adhesion belt
Basal
B
FLAG-
RhoA
empty T19N
DOX - + - +
IB:
kDa 25
20
FLAG
25
20
RhoA
37
GAPDH
C
FLAG-
Rac1
empty T17N
DOX - + - +
IB:
kDa
25
20
FLAG
25
20
Rac1
25
20
Rac1
(Long exposure)
37
GAPDH
D
empty
RhoA T19N
Rac1 T17N
EGFP
E

Fig. S3

(A) Subclone that can block focus expansion (subclone B in Fig. 1B) showed enhanced stress fiber formation compared with subclones that allowed focus expansion. Non-transformed cells (subclone B) were seeded on glass coverslips coated with poly-L-lysine in 12-well plates at a density of 2.0×10^5 cells per well. Cells were cultured for 48 h and then stained with phalloidin. Scale bar = 50 µm.

(B, C) Expression of FLAG-RhoA T19N (B) or Rac1 T17N (C) protein in Tet3G-expressing non-transformed cells (subclone B) was examined using western blotting.

(D, E) Rac1 T17N mutant-expressing non-transformed cells (subclone B) affected focus expansion by *KRASG12D*-expressing cells. (D) Upper: phase-contrast, lower: fluorescence images, scale bar = 500 µm; (E) staining with 0.01% crystal violet, scale bar = 5 mm. Representative data from three experiments are shown.

Supplementary Movies

(A–C) Mixed culture assay was performed with treatment with vehicle (DMSO, A), 1 µM lonidamine (LND, B), or 3 µM domperidone (DPD, C). Images (some are presented in Figs. S1) were acquired every 100 min for approximately 72 h (4300 min). Scale bar = 250 µm.
(D–F) Cell confrontation assay was performed after treatment with DMSO (D), 1 µM LND (E), or 3 µM DPD (F). Images (some are presented in Fig. 7B) were acquired every 10 min for approximately 72 h (4320 min). Scale bar = 250 µm.
